# Characterization of the hepatic flora and metabolome in nonalcoholic fatty liver disease

**DOI:** 10.3389/fmicb.2024.1528258

**Published:** 2024-12-20

**Authors:** Hua Jiang, Hui Wang, Yangfan Guo, Yankun Zhu, Hui Dai, Chenchen Liang, Jianpeng Gao

**Affiliations:** ^1^Shenzhen Ruipuxun Academy for Stem Cell & Regenerative Medicine, Shenzhen, China; ^2^Department of Gastroenterology, Yan’an Hospital Affiliated to Kunming Medical University, Kunming, Yunnan, China; ^3^Central Laboratory of Yan’an Hospital Affiliated to Kunming Medical University, Kunming, China; ^4^Department of Surgery, Yan’an Hospital Affiliated to Kunming Medical University, Kunming, Yunnan, China; ^5^Department of Oncology, Yan’an Hospital Affiliated to Kunming Medical University, Kunming, Yunnan, China

**Keywords:** hepatic microbiota, metabolomics, nonalcoholic fatty liver (NAFL), 16S rDNA sequencing, bacterial DNA profiles

## Abstract

**Background/aim:**

The purpose of this study was to examine the hepatic bacterial composition and metabolome characteristics of patients with NAFLD using 16S rDNA sequencing and metabolomics. The results of the study revealed substantial differences in hepatic bacterial composition and metabolites between the NAFLD group and the control group. These differences were used to identify potential biomarkers that could be employed to diagnose NAFLD.

**Subjects/methods:**

Liver tissues from 13 patients in the NAFLD group and 12 patients in the control group were collected for microbiota examination.

**Results:**

The bacterial DNA profiles of the liver were significantly different between NAFLD patients and controls. NAFLD patients exhibited an enrichment of Enterobacterales, Mycobacteriales, Pseudomonadales, Flavobacteriales and Xanthomonadales, Sphingomonadales, Lysobact, which was characterised by a lack of erales. At the genus level, the abundance values of *Escherichia*-*Shigella*, *Rhodococcus*, and *Chryseobacterium* in the NAFLD group were significantly elevated, while the abundance values of *Stenotrophomonas*, *Lawsonella* and *Sphingobium* were significantly reduced. A total of 402 distinct metabolites were identified between the two groups, with 78 metabolites that were up-regulated and 14 metabolites that were down-regulated. The enrichment of metabolic pathways indicated that linoleic acid metabolism was the most significant contributor to the metabolic differences, and lipid metabolism was substantially differentiated. The hepatic metabolite levels were substantially correlated with the changes in hepatic microflora, as demonstrated by the correlation analysis.

**Conclusion:**

Differences in pathogenesis and host physiological function of NAFLD may be attributed to the hepatic flora and metabolomic characteristics. In the future, this presents new opportunities for the investigation of prospective diagnostic and therapeutic targets for NAFLD.

## Introduction

Non-alcoholic fatty liver disease (NAFLD) has emerged as the most prevalent chronic liver disease and a significant cost to the global health system (Brunt et al., 2015). It is anticipated that the prevalence of NAFLD will increase in tandem with the rise in disorders of glycolipid metabolism, as the progression of NAFLD is closely linked to obesity and insulin resistance. Nevertheless, not much is understood about the pathogenesis of NAFLD. Genetic susceptibility variation, environmental factors, insulin resistance, and alterations in the gut microbiome are believed to be involved in the complex interactions (Arab et al., 2017). The interaction between these factors results in the excessive accumulation of lipids in liver cells and changes in lipid metabolism, which ultimately contribute to the development of NAFLD. In addition, the microbiota is responsible for the regulation of the balance between pro-inflammatory and anti-inflammatory signals, which can result in inflammation and the development of non-alcoholic steatohepatitis (NASH). A progressive form of NAFLD, NASH has the potential to progress to cirrhosis and hepatocellular carcinoma (HCC) and is presently the most common reason for liver transplantation. While there has been consistent progress in the identification of therapeutic targets, pathogenesis, and epidemiology of the disease, the therapeutic area has experienced the most sluggish progress. There are currently no FDA-approved pharmaceuticals to treat this disease, and it is imperative that appropriate therapeutic targets be identified.

Thus, it is imperative to gain a comprehensive understanding of the pathogenesis of NAFLD and the role of the microbiome in its occurrence and development. This knowledge may be beneficial for the diagnosis of the disease, the identification of new therapeutic targets, and the potential for the microbiome to be used as an early clinical warning system for NAFLD. Over the past decade, the gut microbiome has emerged as a significant regulator of substrate metabolism and energy homeostasis in the host. Abnormalities in the structure and, particularly, the function of the microbiota are anticipated to influence the metabolism of the brain, adipose tissue, muscle, and liver. There is a strong correlation between the development of intestinal host-microbial metabolic axes and metabolic diseases and microbial components or metabolites, including lipopolysaccharides, secondary bile acids, dimethylamine, and trimethylamine, as well as compounds produced by carbohydrate and protein fermentation (Macpherson et al., 2016).

In recent years, there has been exploration of the potential mechanisms by which the intestinal microbiota regulates NAFLD. The transfer of harmful microbes and their derived metabolites to the liver through a disrupted intestinal barrier is one of the hypothesized mechanisms. This process results in an inflammatory response in the liver and the co-occurrence of steatosis with dietary factors or metabolite-induced interactions. The notion that gut bacteria affect liver homeostasis is derived from the near anatomical interaction between the gastrointestinal tract and the liver, which is frequently referred to as the “gut-liver axis.” The liver is the initial organ to drain the stomach through the portal vein, which is a critical component of the link between host-microbial interactions. Portal blood contains additional microorganisms that actively or passively migrate from the intestines to the bloodstream, in addition to nutrients. This results in the liver being one of the organs that is most susceptible to gut bacteria and bacteria-derived metabolites (Le Chatelier et al., 2013). Nevertheless, there are limited direct studies on the hepatic microflora, and the precise mechanism of action for the dysregulation of the hepatic microflora that contributes to the development of NAFLD remains unclear. This is expected to result in alterations in the pertinent terminal metabolites in the liver tissues of patients. At present, there is a lack of consensus regarding the specific microorganisms and metabolites present in patients with NAFLD. The identification of specific NAFLD metabolome phenotypes can assist in the development of additional diagnostic tools and therapeutic interventions. Our research directly investigates the microbes and their metabolites in the liver. The basic biological characteristics of microbial composition and metabolomics in the liver tissues of NAFLD patients may offer valuable insights into the disease mechanisms and physiological functions of the host. Consequently, the objective of this investigation was to examine the microbial composition and metabolite characteristics of liver tissue in two distinct coyotes: patients with NAFLD and normal controls. Additionally, the study sought to determine the impact on NAFLD through the regulatory influences between the two.

## Materials and methods

### Recruitment of participants

We enrolled 13 patients (≥18 years) who were newly diagnosed with NAFLD at Yan’an Hospital in Kunming, Yunnan Province, from July 2020 to March 2024. A control group of 12 non-NAFLD subjects, matched by age, sex, and ethnicity, was concurrently recruited. All participants in the control group were asymptomatic volunteers with a standard diet and no recent or chronic illnesses. Liver biopsies were conducted on participants exhibiting abnormal pre-specified imaging criteria, and the biopsies were evaluated blindly, with outcomes determined by the consensus of two expert pathologists. The incidence of NAFLD was ascertained via biopsy. Table 1 presents the demographic information of the subjects enrolled in the study. All subjects granted informed consent to partake in the study. The Medical Ethics Committee of Yan’an Hospital Affiliated to Kunming Medical University approved this study.

**Table 1:** Clinical and biochemical features of patients with NAFLD and the control group.

Variables	Control subjects	NALFD	*p*-value
Number of subjects	12	13	
Female/male (*n*)	5/7	9/3	
Age, years	36.58 ± 8.03	32.00 ± 8.73	0.195
BMI, kg/m^2^	21.45 ± 3.04	38.76 ± 6.45	0.001
Type 2 diabetes (*n*)	0	6	
Fasting plasma glucose, mg/dL	5.71 ± 1.01	6.03 ± 1.86	0.84
Total cholesterol, mg/dL	4.22 ± 0.97	5.98 ± 1.87	0.007
HDL-cholesterol	0.86 ± 0.37	1.08 ± 1.20	0.073
LDL-cholesterol	2.60 ± 0.69	3.25 ± 0.73	0.057
Triglycerides, mg/dL	1.44 ± 0.59	2.11 ± 0.88	0.032
ALT, U/L	23.83 ± 14.82	36.33 ± 29.31	0.371
AST, U/L	25.33 ± 16.34	28.00 ± 20.60	0.073
Degree of steatosis (0–3)	0.58 ± 1.00	1.50 ± 0.67	0.007
Lobular inflammation (0–3)	0	0.42 ± 0.79	0.083
Hepatocellular ballooning (0–2)	0	0.17 ± 0.39	0.148
Fibrosis stage	0	1.33 ± 0.89	0.004
NAFLD activity score (NAS)	0	2.58 ± 3.34	0.008

ALT, serum alanine; AST, aspartate aminotransferase; BMI, body mass index; HDL, high-density lipoprotein; LDL, low-density lipoprotein; NAFLD, non-alcoholic fatty liver disease.

### DNA extraction, 16S rDNA sequencing, and data processing

The FastDNA^®^ Spin Kit for Soil (MP Biomedicals, China) was employed to extract the genomic DNA of liver tissue samples. To assess the purity and integrity of the genomic DNA, it was extracted using 1% agarose gel electrophoresis. Nanodrop 2000 was employed to ascertain the purity and concentration of genomic DNA. The V3–V4 hypervariable region of the 16S r RNA gene was amplified by primers Primer F = Illumina adapter sequence1 + GTGCCAGCMGCCGCGGTAA and Primer R = Illumina adapter for each sample sequence2 + GGACTACHVGGGTWTCTAAT. The Illumina Miseqbenchtop sequencer (Illumina, United States) was employed to sequence the amplified libraries, which were constructed using purified PCR products. A double-terminal sequencing strategy of 2 × 250 bp was employed. In QIIME2 (Bolyen et al., 2019), the original sequencing data underwent quality filtering, noise reduction, splicing, and dechimerization. Feature tables and representative sequences were subsequently generated. Additional analysis was conducted in the DADA2 (v 1.6.0) pipeline (Callahan et al., 2016), and amplicon sequence variants (ASVs) were acquired. The confidence threshold was 0.8, and the RDP classifier algorithm (Wang et al., 2007) was used to compare the taxonomic attribution of ASV representative sequences with the Ribosomal Database Project (RDP) (version 11.5) database. Alpha diversity analysis was implemented to evaluate the diversity and richness of each group. Species, observed, and Chao1 The richness was analyzed using observed species and ACE, while the diversity was analyzed using Shanno, Simpson, InvSimpson, and Coverage. The vegan package from the R project (v2.5.6) was employed to execute the calculations, and the ggplot2 package (v3.3.0) was used to visualize the results. The difference in ASV composition between various samples is measured by beta diversity, which is assessed using principal component analysis (PCA) and principal coordinate analysis (PCoA). Both of these methods are appropriate for the supervised analysis of high-dimensional data. The vegan package (v2.5.6) employs the similarity analysis (ANOSIM) function to determine the importance of beta diversity. Metastats software was employed to compare the microbiota characteristics of healthy controls and NAFLD patients.

### Liver metabolome profile analysis and data preparation

The non-targeted metabolites in liver samples were identified using liquid chromatography-mass spectrometry (LC-MS). Weigh the correct amount of sample in a 2 mL centrifuge tube and add 1,000 μL tissue extract [75% (9:1 methanol: chloroform)]. The steel ball was filled with 25% H_2_O (stored at −20°C) and placed in a tissue grinder. It was ground at 50 Hz for 60 s, ultrasonic at room temperature for 30 min, placed on ice for 30 min, centrifuged, concentrated, and dried. Twenty microliters of each sample was combined into QC samples, while the remaining samples were examined using LCMS. The samples were separated using an ACQUITY UPLC^®^ HSS T3 1.8 μm (2.1 × 100 mm) column (Waters, Milford, MA, United States) at 40°C. Mass spectrometry was carried out at a flow rate of 0.30 mL/min. The mass spectrum data were collected using a Thermo quadrupole-electrostatic field orbital trap high resolution mass spectrometer (Thermo Fisher Scientific, United States) with an electrospray ion source (ESI) that may operate in either positive or negative ion mode. The original data is first converted to mzXML format using MSConvert in the ProteoWizard package (v3.0.8789). In addition, RXCMS was utilized for peak detection, filtering, and alignment. Metabolites are identified using exact mass numbers (<30 ppm) and MS/MS fragmentation patterns, then matched with HMDB, MassBank, LIPID MAPS, mzCloud, and KEGG. To detect differences between groups, we employed orthogonal projection to potential structure discriminant analysis (OPLS-DA) or the discriminant analysis model (PLS-DA). To reduce the risk of overfitting, the model parameters *R*^2^ and *Q*^2^ were calculated to determine the model’s interpretability and predictability. The OPLS-DA model calculates the variable importance in projection (VIP). The *p*-value was calculated using the paired *t*-test in one-dimensional statistical analysis, with p1 serving as the screening criterion for meaningful differential metabolites. Metabolites were annotated using the Kyoto Encyclopedia of Genes and Genomes (KEGG). The route analysis was carried out on the MetaboAnalyst7 database.

Quantitative data conforming to normal distribution were expressed as mean ± SD, and *t*-test was used for comparison between groups. Quantitative data of non-normal distribution were expressed as median (Q1, Q3), and Mann–Whitney *U* test was used for comparison between groups. *p* < 0.05 was considered to be statistically significant. All statistical analyses were performed using the SPSS Statistics 27 software package. *p* < 0.05 was considered significant difference.

## Results

### Baseline characteristics of study participants

Mean age, fasting blood glucose, ALT, AST were not significantly different between the two groups. The number of NAFLD group with type 2 diabetes mellitus, BMI, total cholesterol level, degree of steatosis, lobular inflammation, fibrosis stage, NAFLD activity score (NAS) were significantly higher than the control group. The demographic information of the participants included in the study is shown in Table 1.

### Analysis of liver microbiota diversity

**Figure 1:**
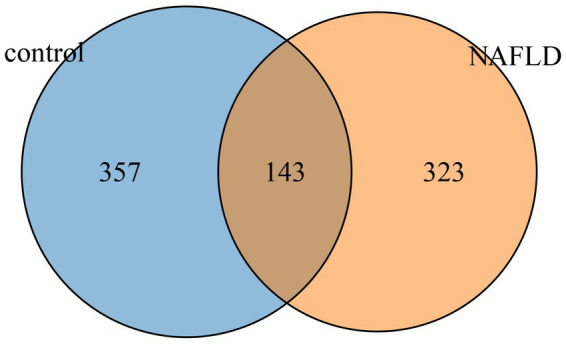
Venn diagram of AVSs from the NAFLD and control groups.

A total of 2,693 high-quality reads were obtained from 25 liver tissue specimen samples. Furthermore, a total of 823 ASVs were obtained. Of these ASVs, 143 were shared among the three groups, while 357 and 323 ASVs were specific to the control and NAFLD groups, respectively (Figure 1).

**Figure 2:**
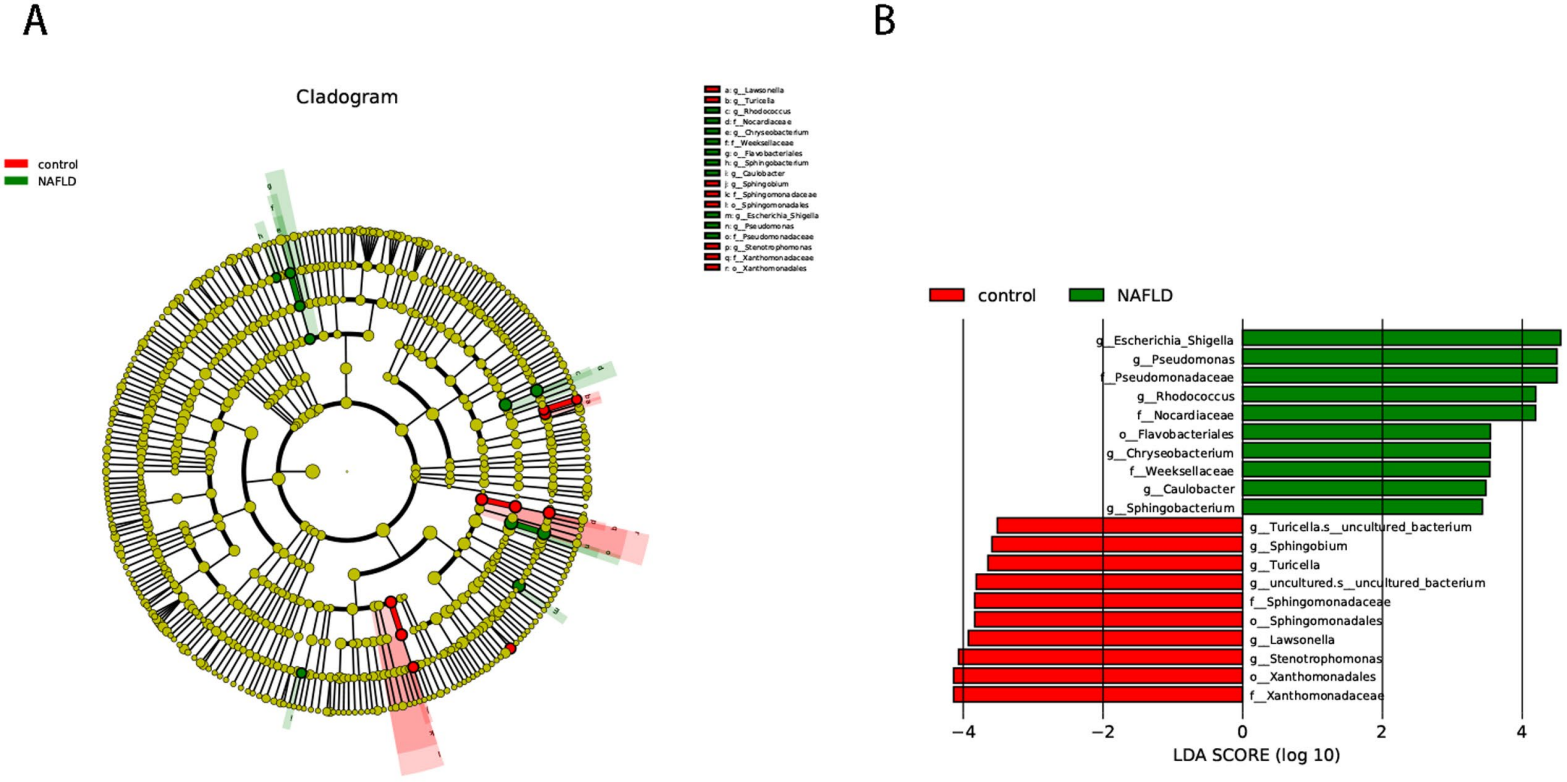
Cladogram representing the LDA-LEfSe results, comparing taxonomic composition of liver bacterial DNA in the samples obtained from the control group and NAFLD patients. **(A)** The top 50 species in each group with the smallest *p*-value are plotted as evolutionary branches. **(B)** The top 10 species in each group with the smallest *p*-value are plotted.

We used the LEfSe approach to find the biometric traits that separate the two sample groups. The LEfSe results confirmed that the bacteria most likely to explain the difference between the two groups are the order_Enterobacterales (genus_*Escherichia*-*Shigella*), order_Mycobacteriales (family_Nocardiaceae, family_Nocardiaceae, genus_*Rhodococcus*), order_Pseudomonadales (family_Pseudomonadaceae, genus_*Pseudomonas*), and order_Flavobacteriales (family_Weeksellaceae, genus_*Chryseobacterium*). The NAFLD group was evidently devoid of the order_Xanthomonadales, order_Sphingomonadales (genus_*Sphingobium*), and order_Lysobacterales (genus_*Stenotrophomonas*) (Figure 2).

**Figure 3:**
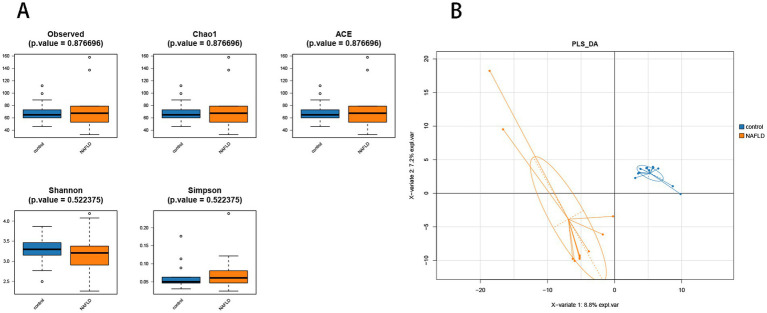
Alpha diversity analysis and beta diversity. **(A)** The comparison of hepatic microbiota alpha diversity between each group, including observed species, Chao1, ACE, Shannon, and Simpson. **(B)** Partial least squares discriminant analysis.

Alpha diversity analysis was performed to determine the richness and diversity of species in each group. The primary metastats used in the Alpha diversity analysis were observed, Chao 1, ACE, Shannon, and Simpson. The results indicated that there was no significant difference in flora richness between the NAFLD group and the control group (*p* > 0.05) (Figure 3A).

PLS-DA analysis showed that there was significant separation of liver microbial community structure between NAFLD and control groups, as shown in Figure 3B. ANOSIM based on UniFrac distances was calculated (*R* = 0.3787, *p* = 0.004).

**Figure 4:**
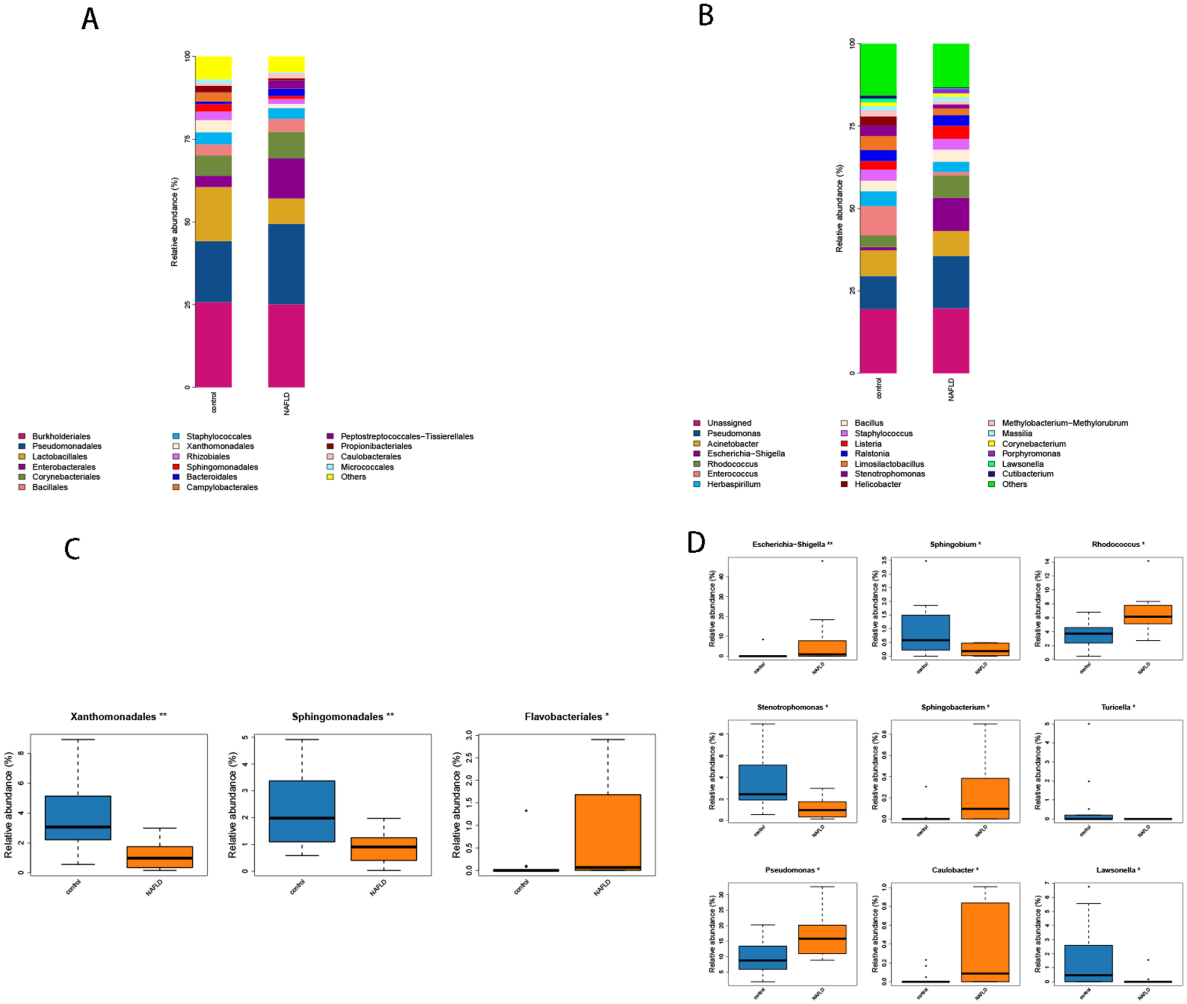
**(A)** Histogram of microbial community composition of each sample at the order level. **(B)** Histogram of microbial community composition of each sample at the genus level. **(C)** Order level difference analysis. **(D)** Analysis of genus level difference.

At the order level, the control group had considerably larger ratios of Lactobacillales (16.34% vs. 7.66%), Xanthomonadales (3.70% vs. 1.25%), and Sphingomonadales (2.25% vs. 0.98%) than the NAFLD group. The NAFLD group had a larger number of Enterobacterales (12.10% vs. 3.42%), Corynebacteriales (8.04% vs. 6.13%), and Bacteroidales (2.07% vs. 0.79%) than the control group (Figure 4A).

The abundance values of Xanthomonadales and Sphingomonadales in the NAFLD group were considerably lower than those in the control group (*p* = 0.004, *p* = 0.008), whereas the abundance values of Flavobacteriales in the NAFLD group were significantly greater than those in the control group (*p* = 0.019) (Figure 4C).

*Escherichia*-*Shigella* (10.07% vs. 0.99%), *Rhodococcus* (6.76% vs. 3.53%), *Enterococcus* (8.89% vs. 1.03%), *Helicobacter* (2.72% vs. 0.07%), and *Pseudomonas* (15.83% vs. 10.05%) were significantly more prevalent in NAFLD than in the control group in terms of genus. *Stenotrophomonas* (3.22% vs. 1.24%), *Lawsonella* (1.06% vs. 0.21%), and *Helicobacter* (2.72% vs. 0.07%) were the most prevalent bacteria in the control group (Figure 4B).

The control group exhibited a substantially lower abundance of *Rhodococcus*, *Escherichia*-*Shigella*, and *Sphingobacterium* than the NAFLD group (*p* = 0.007, *p* = 0.009). The abundance value of *Pseudomonas* (*p* = 0.038) increased considerably, and it was classified as phulum_Pseudomonadota (Figure 4D). Conversely, the abundance values of *Stenotrophomonas*, *Sphingobium*, and *Lawsonella* in the control group were significantly higher than those in the NAFLD group (*p* = 0.014, *p* = 0.011, *p* = 0.031). The abundance of *Turicella* in the phylum actinomycetota and the family Corynebacteriaceae also increased significantly (*p* = 0.017) (Figure 4D).

### Analysis of bacterial metabolites in the liver

**Figure 5:**
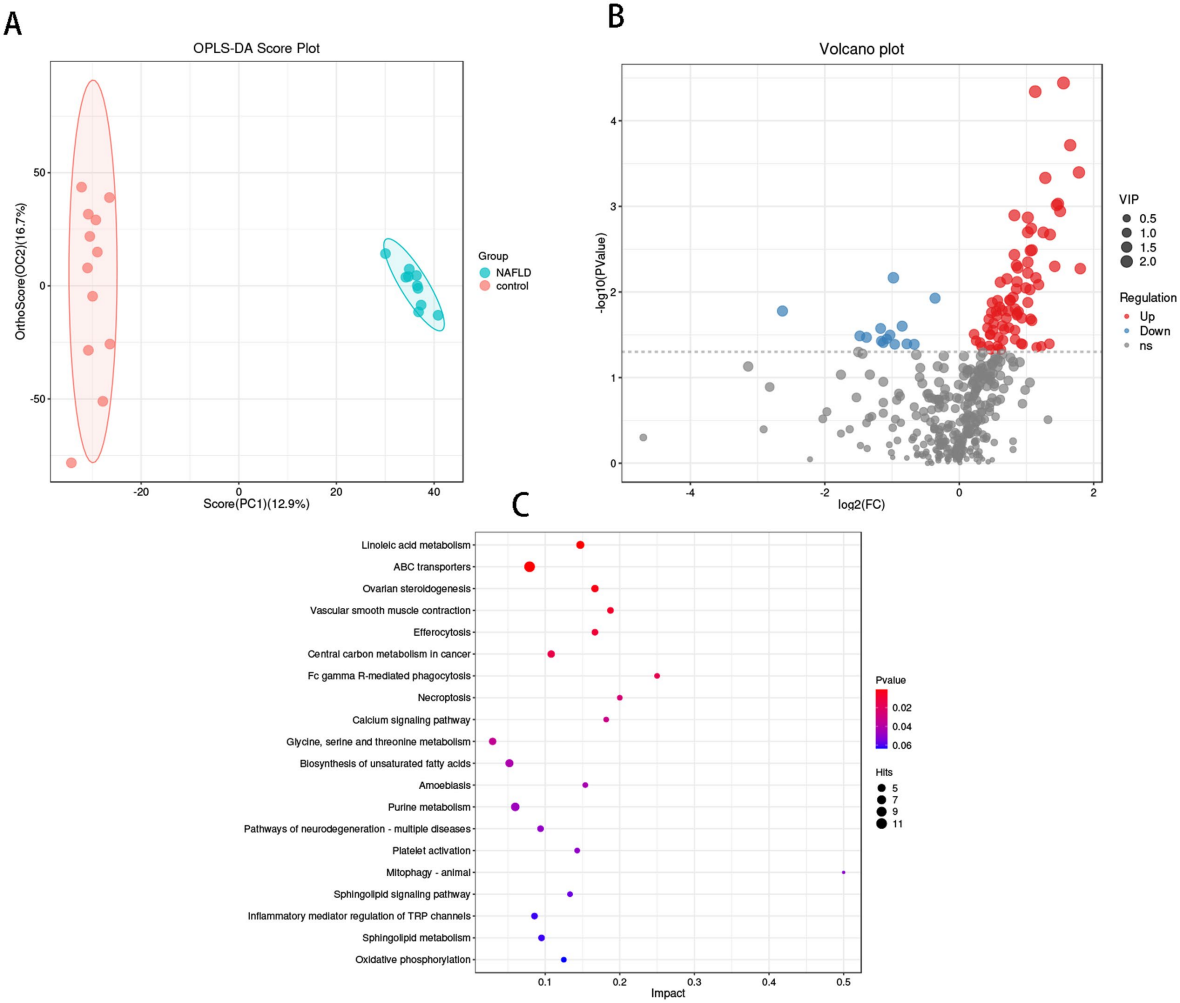
Metabolomics analysis of hepatic flora in control group and NAFLD patients. **(A)** Orthogonal partial least squares discriminate analysis (OPLS-DA) score plots between the control group and NAFLD patients. **(B)** Volcano plot demonstrated metabolites changes in NAFLD compared with control group. **(C)** The bubble plot of KEGG analysis. Bubble charts show the top 20 metabolic pathways that the differentially expressed metabolites enriched in between NAFLD and control groups.

The OPLS-DA results (Figure 5A) demonstrated that the hepatic bacterial flora exhibited differences in metabolite profiles between the NAFLD group and the control group (R2Y = 0.458, Q2Y = 0.994). This suggests that the bacterial metabolites in the liver of NAFLD were transformed, and the metabolic level differences between the two groups could be clearly observed. Four hundred two annotable differential metabolites were screened from the NAFLD group and control group using VIP >1 and *p* < 0.05 as the first principal component of the OPLS-DA model. This included 78 up-regulated metabolites and 14 down-regulated metabolites (Figure 5B). Five metabolites with significant differences in up-regulation and down-regulation between the NAFLD group and the control group were evaluated after further adjusting the screening conditions (Table 2). The majority of them are carboxylic acids and their derivatives, steroids and steroid derivatives, azagands and complexes, fatty acyl groups, fatty acids and conjugates, linolenic acid metabolism, nucleotide metabolism, glycerophospholipids, and glutathione synthase. Significant differential metabolism is observed in the lipid metabolism of these pathways (Figure 5B).

**Table 2:** Intrahepatic differences in metabolites between NAFLD patients and controls.

No.	ID	Metabolite	VIP	*p*-value	Metabolite changes
1	M188T43	N1-Acetylspermidine	3.61701 × 10^−5^	3.61701 × 10^−5^	Up
2	M269T488	Dehydroepiandrosterone	2.123851592	4.56635 × 10^−5^	Up
3	M183T243	2,4-Dinitrophenol	1.965019809	0.00019294	Up
4	M442T39	GDP	1.965874703	0.000401213	Up
5	M327T589	Docosahexaenoic acid	1.934366578	0.000464984	Up
6	M307T54	Glutathione	1.602143705	0.006818564	Down
7	M361T477	2-Arachidonoylglycerol	1.410222229	0.011814339	Down
8	M424T585	Lupenone	0.429842898	0.01667075	Down
9	M130T56	Pipecolic acid	0.026654679	1.326694286	Down
10	M218T317	N-Acetylserotonin	0.02503084	1.394832999	Down

The metabolic pathway enrichment results were derived from the KEGG pathway database. The findings indicated that the primary metabolite enrichment pathways were linoleic acid metabolism, ABC transporter, phagocytosis, necrotic apoptosis, and calcium signaling pathways. Linoleic acid metabolism was the most significant contributor to metabolic differences (Figure 5C).

### Correlation analysis of differential flora and metabolites related to NALFD

**Figure 6:**
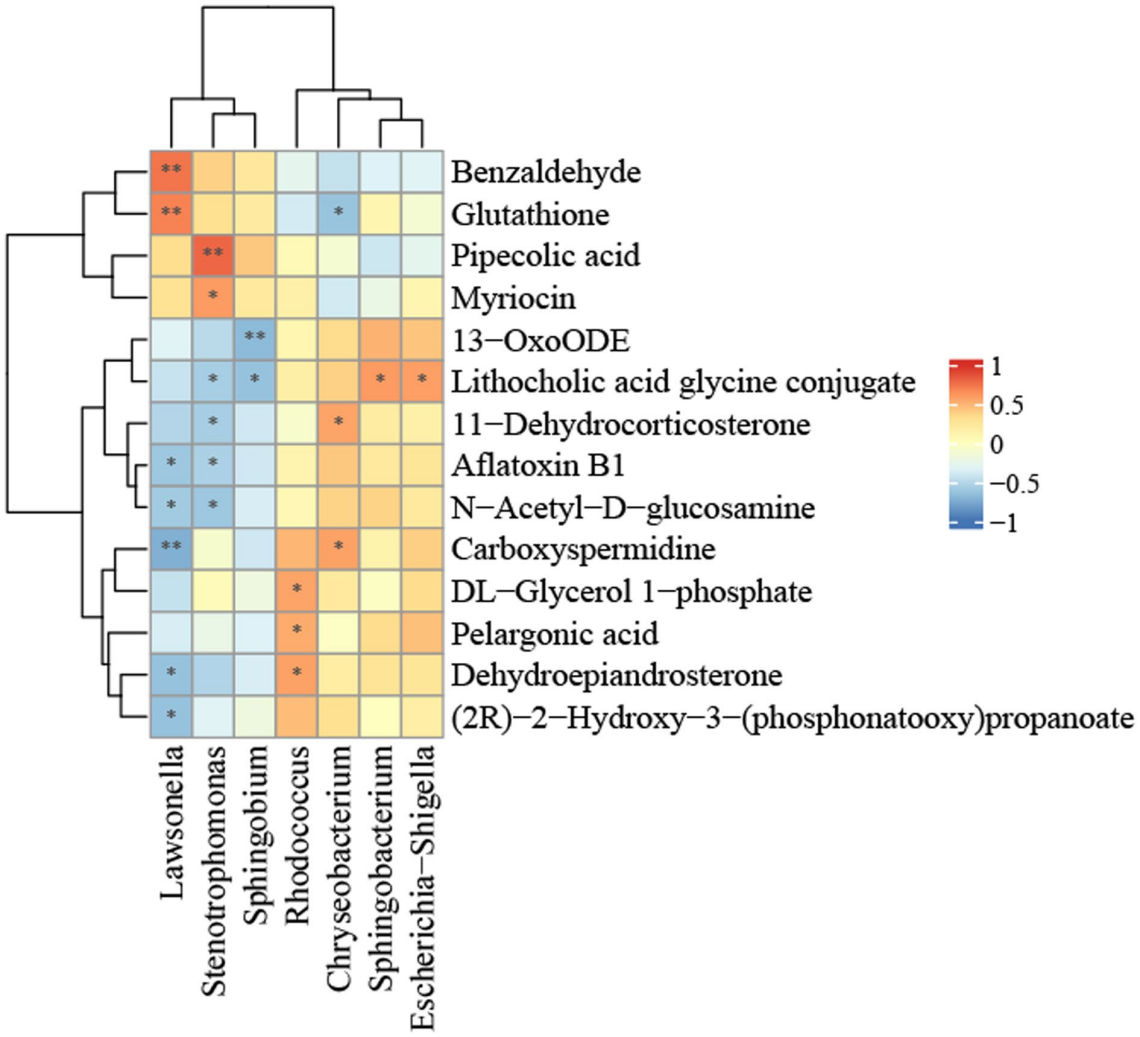
Heatmap of the correlation between bacterial metabolites and bacterial relative abundance in the NALFD liver. ^*^p < 0.05, ^**^p < 0.01, ^***^p < 0.0001, and ^****^p < 0.0001.

The relationship between hepatic flora and metabolite groups was analyzed using Pearson correlation analysis. In order to investigate potential sources of metabolites in the liver, we conducted a generic-level analysis of the correlations between the liver flora and metabolites. It was determined that the bacteria *Lawsonella*, *Stenotrophomonas*, and *Sphingobium*, which are abundant in the liver of the control group, and the bacteria *Rhodococcus*, *Chryseobacterium*, and *Escherichia*-*Shigella*, which are significantly more abundant in the liver of NAFLD patients, have a strong correlation with differential metabolites, as illustrated in Figure 6. *Lawsonella* was positively correlated with glutathione and benzaldehyde, and negatively correlated with carboxyspermidine, (2R) -2-hydroxy-3-(phosphonatooxy)propanoate. Pipecolic acid and myriocin were positively correlated with *Stenotrophomonas*. 13-oxoODE is negatively correlated with *Sphingobium*, while lithocholic acid glycine conjugate is positively correlated with *Escherichia*-*Shigella* and *Sphingobacterium* and negatively correlated with *Sphingobium* and *Stenotrophomonas*. *Rhodococcus* was positively correlated with dehydroepiandrosterone. *Chryseobacterium* and *Lawsonella* were positively and negatively correlated with carboxyspermidine.

## Discussion

There is increasing interest in elucidating the microbiome’s role in the pathophysiology of MAFLD, with numerous gut bacterial communities identified in various studies as components of microbial patterns in NAFLD. Intestinal flora significantly influences health, and its imbalance is associated with the expedited advancement of NAFLD. Intestinal bacteria and their metabolites directly access the liver via the portal vein and indirectly influence the onset and progression of NAFLD, either directly or through signaling pathways mediated by their constituents (Fang et al., 2022). Our research directly examined the microbiota in human liver tissue, minimizing the confounding influence of intestinal microflora, with the diagnosis of NAFLD relying on liver imaging and biopsy.

We concentrated on NAFLD and the control group, demonstrating that the bacterial DNA signature in the liver of NAFLD is greatly influenced by the host phenotype. The hepatic bacterial community composition in the two groups was equivalent at many levels; nevertheless, substantial variations were noted in abundance analysis, diversity measurement, and the predictive utility of bacterial DNA. Distinct metabolites in the samples from the two groups influenced the onset and progression of NAFLD, and we additionally identified the association between the varying bacterial communities and metabolites.

Nonetheless, individuals with NAFLD (validated by liver needle biopsy) exhibited no significant differences in the Metastats analysis of α diversity in liver bacteria when compared to controls without NAFLD. In contrast, other researchers (Monga Kravetz et al., 2020) reported diminished bacterial diversity in NAFLD subjects, potentially attributable to the sample size in our study. In our study, the disparity between the two groups was substantial, although the variance within the two groups was minimal. Consequently, a notable disparity in the amount of hepatic flora existed between the two groups at varying levels. The prevalence of Enterobacteriales, Corynebacteriales, and Flavobacteriales in the NAFLD group, together with *Escherichia*-*Shigella*, *Rhodococcus*, and *Chryseobacterium* in the NAFLD group, was significant. The levels were markedly elevated compared to the control group, and were devoid of Lactobacillales, Xanthomonadales, Sphingomonadales, *Stenotrophomonas*, *Lawsonella*, and *Sphingobium*, which constituted a considerably smaller proportion than in the control group. This may facilitate the progression of the condition. *Escherichia*-*Shigella* has been shown to induce steatohepatitis and fibrosis in non-obese rats through the secretion of msRNA 23,487 (Xin et al., 2022). *Escherichia*-*Shigella* is linked to steatosis and necrotic inflammatory activity, whereas *Shigella* is related with fibrosis and necrotic inflammatory activity (Cornejo-Pareja et al., 2024). *Rhodococcus* is intimately associated with the phenotype of NAFLD and can effectively differentiate between NAFLD patients and healthy non-NAFLD individuals (Yang et al., 2024). *Chryseobacterium* is a non-fermentative gram-negative bacterium. It is a conditional pathogen that remains non-infectious under typical conditions but may induce infection when the immune system is compromised. Flavobacteriaceae and Porphyromonadaceae have markedly proliferated in the intestines of animals subjected to a high-fat diet, although have not been documented in human intestines (Chen and Vitetta, 2020). Conversely, *Stenotrophomonas* rectified ecological imbalances in individuals with NAFLD, stabilized inflammatory cytokine expression and mucosal immune function, and mitigated NAFLD and its associated hazards (Ayob et al., 2023). *Lawsonella* participates in the metabolic pathways of fatty acids, nucleotides, and carbohydrates. Bacteroidetes and bacilobacteria are believed to significantly contribute to intestinal homeostasis, comprising over 90% of the bacteria present in healthy human intestines (Wu and Fan, 2023), a finding corroborated by our research results. NAFLD patients have a deficiency in the protective effects of beneficial bacteria, which are essential for combating inflammation and stabilizing hepatic immunity, hence exposing the liver to bacteria that readily induce immune suppression and inflammatory responses.

Analysis revealed that bacterial metabolites in the liver of both groups were highly enriched, with a notable difference between the two groups. N1-Acetylspermidine, an acetyl-derivative of polyamines, is up-regulated in the liver of NAFLD patients and may serve as a valuable biomarker linked to the course of nonalcoholic fatty liver disease (Chai et al., 2023). Irregular steroids and steroid derivatives dehydroepiandrosterone may influence the progression of NAFLD and engage in lipid metabolism, however the function of its signaling in the pathogenesis of NAFLD is not yet elucidated (Lützhøft et al., 2024). 13-oxoODE is an oxidized lipid derivative of linoleic acid (LA) and correlates with the histological severity of NAFLD (Langner et al., 2022). GDP participates in the metabolism of fructose and mannose. Conversely, glutathione, a down-regulated metabolite, has the potential to ameliorate NAFLD (Rom et al., 2020). The metabolic route exhibiting the most significant variation is linoleic acid metabolism, a process of fatty acid synthesis and degradation, which regulates blood glucose levels and facilitates the oxidation of saturated fatty acids while diminishing the synthesis of cholesterol and triacylglycerol. To elucidate the pathogenicity of metabolites, it is essential to comprehend the bacterial origins of these metabolites and their interrelationships.

To further investigate the bacterial origins of metabolites in the liver, we performed a comprehensive examination of both bacteria and metabolites within the liver. *Lawsonella* is classified within the phylum Actinomycetota, class Actinomycetes, order Mycobacteriales, and family Lawsonellaceae. Benzaldehyde, which has a positive correlation, inhibits fat formation in normal human liver cells (Li et al., 2021) and reduces the onset of NAFLD, potentially linked to the metabolic product aldehyde oxidase 2. Furthermore, glutathione is favorably correlated with the production of glutathione synthetase, which plays a role in glutathione metabolism, and the synthesis of glutathione mitigates NAFLD (Rom et al., 2020). *Lawsonella* exhibits an inverse correlation with the metabolite (2R)-2-hydroxy-3-(phosphonatooxy)propanoate ethyl propionate, which is found in all eukaryotes, ranging from yeast to humans. Ethyl propionate is connected with various known ailments, including autism, irritable bowel syndrome, ulcerative colitis, and non-alcoholic fatty liver disease. Furthermore, it has been linked to congenital metabolic problems, such as celiac disease. Ethyl propionate, a volatile organic molecule, has been recognized as a fecal biomarker for *C. difficile* infection (Patel et al., 2019).

*Stenotrophomonas* is classified within the phylum Pseudomonadota, class Gammaproteobacteria, order Lysobacterales, and family Lysobacteraceae. Ecological disturbances in NAFLD patients can be ameliorated by stabilizing the expression of inflammatory cytokines and enhancing mucosal immune function (Ayob et al., 2023). The former had a favorable correlation with pipecolic acid and myriocin, respectively. Metabolomic studies of serum and liver indicated that the former contained a non-coding amino acid. The study findings demonstrated that early consistent exercise may improve the anti-inflammatory immune response in middle-aged male mice via epigenetic modulation of immune metabolism. The hepatic production of pipecolic acid is pivotal (Vlasova et al., 2022), being intricately linked to fatty acid synthase and fatty acid desaturase, and constitutes a significant component of the lipid metabolism route. Insufficient pipecolic acid can result in fatty acid oxidation disorder, bile acid synthesis defect, and long-chain fatty acid transport deficiency, culminating in lipid metabolism problem. The latter suppressed ceramide and lipid buildup while enhancing fibrosis in liver tissue samples from rats subjected to a high-fat diet (HFD), and myriocin also markedly ameliorated liver inflammation and apoptosis in HFD rats (Fu et al., 2021).

*Sphingobium* is classified under phylum Pseudomonadota, class Alphaproteobacteria, order Sphingomonadales, and family Sphingomonadaceae. The negatively correlated 13-oxoODE, an oxidized lipid derivative of linoleic acid, correlates with the histological severity of NAFLD and facilitates the evolution of NASH by elevating oxidized fatty acids (22505276) (Zein et al., 2012).

*Rhodococcus* is classified within the phylum Actinomycetota, class Actinomycetes, order Mycobacteriales, and family Nocardiaceae. Aberrant synthesis and metabolism of positively linked substances dehydroepiandrosterone and catecholamines may be linked to the onset of NAFLD (Papanastasiou et al., 2017). Levels of 16 hydroxydehydroepiandrosterone sulfate (16-OH-DHEA-S) elevated with the advancement of fibrosis (Tokushige et al., 2013).

*Chryseobacterium* is classified under phylum Bacteroidota, class Flavobacteriia, order Flavobacteriales, and family Weeksellaceae. Carboxyspermidine, positively associated with *Chryseobacterium*, serves as a novel biomarker for NAFLD progression, with elevated levels correlating with the condition (Coelho et al., 2022). 11-Dehydrocorticosterone, which is positively correlated with metabolic syndrome (Harno et al., 2013), exhibits a substantial association with NAFLD and a negative correlation with glutathione.

The glycine conjugate of lithocholic acid is elevated in the intestines of patients with NAFLD (Jiao et al., 2018), potentially linked to fatty acid oxidation dysfunction and positively connected with *Escherichia*-*Shigella* and *Sphingobacterium*. It had a negative correlation with *Sphingobium* and *Stenotrophomonas*. Phylum Bacteroidota, class Sphingobacteriia, order Sphingobacteriales, family Sphingobacteriaceae *Escherichia*-*Shigella* is classified within the phylum Pseudomonadota, class Gammaproteobacteria, order Enterobacterales, and family Enterobacteriaceae.

This work used multi-omics to connect hepatic microbiota and metabolites. Correlation analysis indicated that the liver microbiota not only modulates inflammation and immunity but also regulates lipid synthesis, metabolism, and transport via associated metabolites, influences hepatic fat accumulation, and significantly impacts the enhancement or exacerbation of inflammation and fibrosis. This study highlights that metabolic disorders resulting from bacterial imbalance in the liver are significant contributors to the pathogenesis of NAFLD, and investigating the relationship between specific metabolites and bacterial flora may ultimately aid in regulating bacterial flora function in NAFLD treatment.

This study has certain drawbacks. The limited sample size necessitates external validation via larger samples and multi-center experiments. However, confounding variables can be efficiently managed by enlisting healthy participants matched by age, gender, and ethnicity. This study was a case–control study. Although our data indicate a functional relationship among the bacteriome, metabolome, and illness, causality remains undetermined, and the mechanism behind this functional correlation requires additional investigation.

In conclusion, examining the correlation between the human hepatic microbiota and NAFLD reveals distinct bacterial communities and metabolic traits, hence presenting new opportunities for researchers to investigate the possibly advantageous effects of specific nutrient supplementation. This study establishes an experimental foundation for developing prospective diagnostic and therapeutic targets in the future.

## Data Availability

The datasets presented in this study can be found in online repositories. The names of the repository/repositories and accession number(s) can be found in the article/supplementary material.
