# Corrigendum: Characterization of the hepatic flora and metabolome in nonalcoholic fatty liver disease

**DOI:** 10.3389/fmicb.2025.1591065

**Published:** 2025-04-23

**Authors:** Hua Jiang, Hui Wang, Yangfan Guo, Yankun Zhu, Hui Dai, Chenchen Liang, Jianpeng Gao

**Affiliations:** ^1^Shenzhen Ruipuxun Academy for Stem Cell & Regenerative Medicine, Shenzhen, China; ^2^Department of Gastroenterology, Yan'an Hospital Affiliated to Kunming Medical University, Kunming, Yunnan, China; ^3^Central Laboratory of Yan'an Hospital Affiliated to Kunming Medical University, Kunming, China; ^4^Department of Surgery, Yan'an Hospital Affiliated to Kunming Medical University, Kunming, Yunnan, China; ^5^Department of Oncology, Yan'an Hospital Affiliated to Kunming Medical University, Kunming, Yunnan, China

**Keywords:** hepatic microbiota, metabolomics, nonalcoholic fatty liver (NAFL), 16S rDNA sequencing, bacterial DNA profiles

This Corrigendum addresses an error in the **author affiliations** section of the originally published article. In the published article, the affiliation “Shenzhen Ruipuxun Academy for Stem Cell & Regenerative Medicine” was omitted.

The author list and affiliations in the published article were listed as:

Hua Jiang^1^, Hui Wang^1*^, Yangfan Guo^2^, Yankun Zhu^3^, Hui Dai^4^, Chenchen Liang^1^ and Jianpeng Gao^1*^

^1^Department of Gastroenterology, Yan'an Hospital Affiliated to Kunming Medical University, Kunming, China

^2^Central Laboratory of Yan'an Hospital Affiliated to Kunming Medical University, Kunming, China

^3^Department of Surgery, Yan'an Hospital Affiliated to Kunming Medical University, Kunming, China

^4^Department of Oncology, Yan'an Hospital Affiliated to Kunming Medical University, Kunming, China

The corrected affiliations and author list appear below:

Hua Jiang^2^, Hui Wang^2*^, Yangfan Guo^3^, Yankun Zhu^4^, Hui Dai^5^, Chenchen Liang^2^ and Jianpeng Gao^1, 2*^

^1^Shenzhen Ruipuxun Academy for Stem Cell & Regenerative Medicine, Shenzhen, China

^2^Department of Gastroenterology, Yan'an Hospital Affiliated to Kunming Medical University, Kunming, Yunnan, China

^3^Central Laboratory of Yan'an Hospital Affiliated to Kunming Medical University, Kunming, China

^4^Department of Surgery, Yan'an Hospital Affiliated to Kunming Medical University, Kunming, Yunnan, China

^5^Department of Oncology, Yan'an Hospital Affiliated to Kunming Medical University, Kunming, Yunnan, China

The authors apologize for this error and state that this does not change the scientific conclusions of the article in any way. The original article has been updated.

